# Occurrence, Sources, and Ecological Risks of Organochlorine Pesticides in Sediments of Typical Plateau Lakes, Southwest China

**DOI:** 10.3390/toxics14070556

**Published:** 2026-06-25

**Authors:** Zhonghong Zhao, Li Bao, Min Ye, Naiming Zhang

**Affiliations:** 1 College of Plant Protection, Yunnan Agricultural University, Kunming 650201, China; 2 College of Resources and Environment, Yunnan Agricultural University, Kunming 650201, China; 3 Yunnan Soil Fertility and Pollution Restoration Laboratory, Yunnan Agricultural University, Kunming 650201, China; 4 The Research Center for Smart Greenhouse Agriculture Engineering of Yunnan Provincial Universities, Yunnan Agricultural University, Kunming 650201, China

**Keywords:** plateau lakes, organochlorine pesticides, sediment contamination, source apportionment, ecological risk assessment

## Abstract

This study investigated the contamination characteristics, sources, and ecological risks of organochlorine pesticides (OCPs) in surface sediments from three plateau lakes in southwestern China (Qilu Lake, Dianchi Lake, and Yangzonghai Lake). Significant differences in OCP pollution levels were observed among the three lakes. Hexachlorocyclohexanes (HCHs) were identified as the dominant contaminants, reflecting historical technical HCH input and subsequent long-term aging, whereas dichlorodiphenyltrichloroethanes (DDTs) exhibited generally low concentrations and originated primarily from historical technical use, with predominantly aerobic degradation. Principal component analysis (PCA) revealed that agricultural non-point source pollution was the main contributor to OCP residues. Ecological risk assessment demonstrated that most OCPs posed low or negligible risk; however, γ-HCH (lindane) ubiquitously presented moderate risk across all lakes, with one site exceeding the high-risk threshold. Endrin derivatives and methoxychlor further elevated combined risks at specific sites. Notably, the unique hydrological characteristics of plateau lakes may enhance OCP retention and accumulation in sediments. These findings provide a scientific basis for ecological risk management and pollution control in plateau lakes.

## 1. Introduction

Organochlorine pesticides (OCPs), as typical persistent organic pollutants (POPs), have become a focal issue in the field of global environmental science due to their high toxicity, environmental persistence, long-range transport potential, and bioaccumulation capability [1,2]. Although most OCPs, including dichlorodiphenyltrichloroethane (DDT) and hexachlorocyclohexane (HCH), have been banned or strictly restricted worldwide for decades, their chemical stability and slow degradation have led to their widespread detection in soil, water, sediments, and biological matrices [2,3,4,5]. Sediments serve as an important “sink” for OCPs in lake ecosystems, not only recording the history of regional pesticide use but also potentially acting as secondary pollution sources under certain conditions, posing potential ecological risks to overlying water and benthic organisms [6,7].

International research on OCPs contamination in lake sediments has become relatively extensive [3,8,9,10,11,12,13]. These studies have demonstrated that the residual levels, compositional characteristics, and ecological risks of OCPs are closely related to agricultural activity intensity, degree of industrialization, climatic conditions, and hydrological environments within the watersheds. Domestically, relevant research has mainly focused on eastern plain lakes (e.g., Taihu Lake, Chaohu Lake, Honghu Lake) and the Yangtze River Basin [5,7,14,15,16]. For instance, studies have found that the relatively high residues of HCHs and DDTs in the sediments of Taihu Lake are primarily associated with historically intensive agricultural use and industrial inputs in the watershed [7]. Meanwhile, studies on Chaohu Lake and Honghu Lake have revealed the response of OCP compositional patterns to regional pesticide use histories [5,15]. These studies have laid an important foundation for understanding the patterns of OCP contamination in lakes under the influence of human activities.

However, compared with eastern plain lakes, plateau lakes possess unique characteristics such as closed watersheds, relatively low water temperatures, weaker microbial degradation capacity, and more stable sedimentary environments. Consequently, the environmental behavior and ecological effects of POPs in plateau lakes may exhibit significantly different features [6,17,18]. In recent years, some plateau lakes (e.g., Dianchi Lake and Erhai Lake) have faced the dual pressures of eutrophication and emerging contaminants [19,20]. Nevertheless, systematic regional comparative studies on OCP contamination in the sediments of southwestern plateau lake clusters remain relatively limited. In particular, there is a notable lack of cross-lake comparisons and mechanistic investigations regarding differences in pollution levels, source characteristics, and ecological risks among these lakes.

The Yunnan Plateau lake cluster (including Qilu Lake, Dianchi Lake, and Yangzonghai Lake) is located on the Yunnan-Guizhou Plateau and represents an important freshwater resource and ecological barrier for the region. Although the three lakes share a similar climatic zone, significant differences exist in the types and intensities of human activities within their watersheds: the Qilu Lake watershed is dominated by vegetable and flue-cured tobacco cultivation, with prominent agricultural non-point source pollution [21,22]; Dianchi Lake simultaneously bears the dual pressures of urbanization and agricultural activities [23,24]; Yangzonghai Lake was historically affected by industrial pollution events [18,25]. These differences provide an ideal study area for comparative research on the pollution characteristics of OCPs in plateau lake sediments under different intensities of human activity. Although sporadic reports have addressed pesticide residues in the water bodies and sediment cores of individual lakes [17,18,26,27], no study has yet simultaneously investigated Qilu Lake, Dianchi Lake, and Yangzonghai Lake for a systematic comparative analysis of 20 OCPs using uniform sampling periods, identical analytical methods, and consistent quality control procedures. Consequently, the driving factors for differences in OCP residual levels among lakes, the similarities and differences in their sources, and the resulting ecological risk classification all lack systematic comparative investigation.

Based on this, this study takes Qilu Lake, Dianchi Lake, and Yangzonghai Lake on the Yunnan Plateau as research subjects. Through field sampling and laboratory analysis, the residual concentrations of 20 OCPs (including HCHs, DDTs, chlordanes, drins, endosulfans, etc.) in surface sediments were systematically determined. The specific research objectives are: (1) to reveal the pollution levels, compositional characteristics, and regional differences in OCPs in the surface sediments of the three lakes, and to compare them with other water bodies both domestically and internationally; (2) to identify the main sources of OCPs using diagnostic ratio analysis and principal component analysis (PCA), distinguishing between historical residues and fresh inputs; (3) to assess the potential ecological risks of individual and combined OCPs to benthic ecosystems using the risk quotient (RQ) method, and to identify key risk compounds and high-risk sites. The findings will provide a scientific basis for understanding the environmental behavior of persistent organic pollutants in plateau lakes, managing ecological risks, and implementing coordinated governance of agricultural and industrial activities within the watersheds.

## 2. Materials and Methods

### 2.1. Study Area and Sampling

Three fault-controlled plateau lakes were selected. Dianchi Lake is the largest plateau lake in Yunnan Province, with an average depth of ~5 m. Long-term urban expansion and industrial/agricultural pollution caused severe eutrophication. After years of comprehensive treatment, water quality has reached Class IV (GB 3838-2002) [28], though ecological restoration remains challenging [29]. Yangzonghai Lake has an average depth of ~20 m, making it a typical deep mesotrophic lake. Following the 2008 arsenic contamination incident, remediation has been successful: water quality is now Class III (GB 3838-2002), and aquatic ecology is improving [25]. Qilu Lake has an average depth <4 m. Its basin is a core area for high-altitude specialty vegetables. Long-term intensive agricultural non-point source pollution led to persistently inferior Class V (GB 3838-2002) water quality [30]. Although the lake achieved annual de-eutrophication in 2025, ecological fragility remains high.

**Figure 1:**
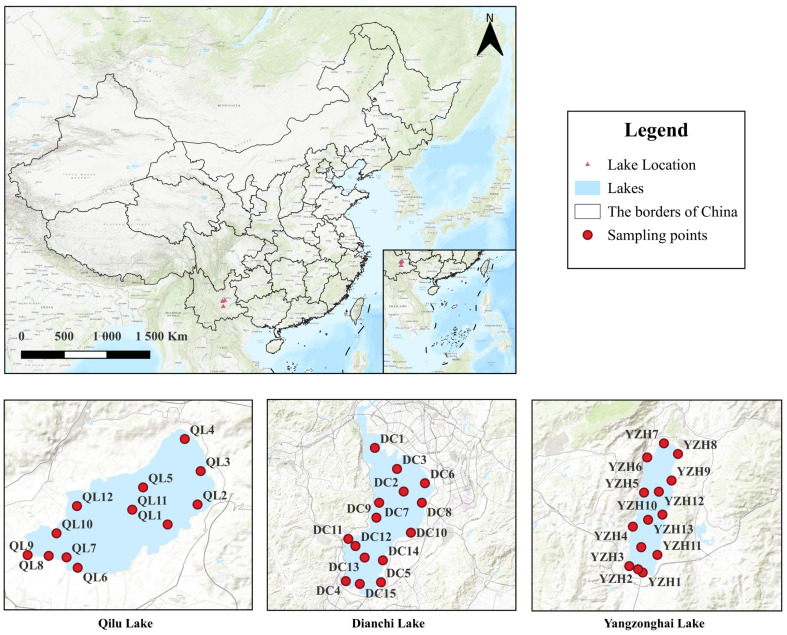
Sampling site locations in three plateau lakes.

In 2023, surface sediment samples were collected from the three lakes (sampling sites shown in Figure 1), with a total of 41 sampling sites (12 sites in Qilu Lake, 15 sites in Dianchi Lake, and 14 sites in Yangzonghai Lake). A grid sampling strategy combined with water depth gradients was employed to ensure coverage of different hydrological zones, including inlet river mouths, lake centers, littoral zones, and outlets. At each site, surface sediments (0–15 cm) were collected using a Petersen grab sampler, and three grabs were mixed to form one composite sample. Three samples were taken near each point, homogenized, and placed into brown glass bottles with polytetrafluoroethylene liners pre-washed with 40 mL n-hexane and distilled water. Samples were transported to the laboratory within 12 h and stored frozen at −20 °C. Prior to analysis, samples were freeze-dried, ground with a ceramic mortar, passed through a 200 mesh sieve, and stored in aluminum foil bags in the dark.

### 2.2. Sediment Property Analysis

Total organic carbon (TOC) in sediments was determined by TOC auto-analyzer [31,32,33]. Total nitrogen (TN) was measured using the alkaline potassium persulfate digestion–ultraviolet spectrophotometric method [34]. Total phosphorus (TP) was determined following the Standard Measurement and Test (SMT) protocol, and phosphorus in the extracts was quantified by the molybdenum blue method at 880 nm [35,36].The analytical results of TOC, TN, and TP in surface sediments from the three lakes are presented in Figure S1.

### 2.3. Pretreatment of OCP Samples

Freeze-dried sediment samples (mixed with 8 g of activated quartz sand, activated at 450 °C for 4 h) were extracted using accelerated solvent extraction (ASE 350, Dionex, Thermo Fisher Scientific, Sunnyvale, CA, USA) with dichloromethane. The extract was concentrated by rotary evaporation and purified on a silica gel-alumina column (2:1). Target compounds were eluted with n-hexane/dichloromethane (7:3, *v*/*v*). After solvent exchange and further concentration, the final extract was adjusted to 1 mL and stored at −20 °C until analysis.

### 2.4. Instrumental Analysis of OCPs

Quantification was performed using a gas chromatograph (Agilent 7890A, Santa Clara, CA, USA) equipped with a ^63^Ni micro-electron capture detector (μ-ECD). Separation was achieved on an HP-5MS capillary column (30 m × 0.25 mm × 0.25 μm). Helium was used as the carrier gas at a constant flow rate of 1.5 mL/min. High-purity nitrogen (60 mL/min) served as makeup and auxiliary gas. Injector temperature: 250 °C; detector temperature: 320 °C. A 1-μL sample was injected in splitless mode. Peak identification was confirmed using an Agilent GC-MS in full scan (*m*/*z* 45–500) and selected ion monitoring (SIM) modes.

### 2.5. Quality Assurance and Quality Control (QA/QC)

OCP peaks were identified by retention times and spectra of standard solutions. Quantification used an external standard-internal standard method with 2,4,5,6-tetrachloro-m-xylene as the internal standard. Calibration curves (concentration vs. peak area) were established with seven serial dilutions; coefficients of determination (R^2^) ranged from 0.992 to 0.999. Six repeated injections of the same standard gave relative standard deviations (RSDs) of 0.58–6.93%. Standard solutions were spiked every 10 samples to recalibrate retention times.

Method detection limits (MDLs) were determined as the analyte amount producing a signal-to-noise ratio (S/N) of 3 in a matrix sample, ranging from 0.003 to 0.086 ng/g. Laboratory blanks showed no interfering contaminants. Spiked recoveries of OCP congeners using diatomaceous earth as a surrogate sample ranged from 79 to 110%, meeting USEPA quality requirements for environmental sample analysis.

The 20 target OCPs were: α-HCH, β-HCH, γ-HCH, δ-HCH, Heptachlor, Aldrin, Heptachlor epoxide, γ-chlordane (trans-chlordane), α-chlordane (cis-chlordane), α-endosulfan, p,p’-DDE, Dieldrin, Endrin, β-endosulfan, p,p’-DDD, Endrin aldehyde, Endosulfan sulfate, p,p’-DDT, Endrin ketone, and Methoxychlor. The mixed standard was purchased from Supelco (Bellefonte, PA, USA). All organic reagents (dichloromethane, isopropanol, n-hexane) were of chromatographic grade.

Recoveries for the 20 OCPs ranged from 69% to 101%, with lower recoveries for p,p’-DDT (69%) and methoxychlor (71%) possibly leading to slight underestimation. Detection limits met trace analysis requirements.

### 2.6. Ecological Risk Assessment for Sediment OCPs

The risk quotient (*RQ*) method based on the EU Technical Guidance Document (EU-TGD) was adopted to assess the ecological risks of OCPs in sediments. In this study, porewater concentrations were not directly measured but were derived from sediment concentrations using the equilibrium partitioning method, as recommended by the EU-TGD. A total of 41 sediment samples were collected, from which 41 corresponding porewater concentration values were derived.

The *RQ* is calculated as:

$$RQ=\frac{C_{pore}}{PNEC}$$

where *C*_pore_ is the sediment porewater concentration (ng·L^−1^) derived from the equilibrium partitioning method, and *PNEC* is the predicted no-effect concentration in water (ng·L^−1^). *C*_pore_ is calculated as:

$$C_{pore}=\frac{C_{s}}{K_{oc}\times f_{oc}}$$

where *C*_s_ = measured concentration in sediment (ng·g^−1^ dry weight), *K*_oc_ = organic carbon-normalized partition coefficient (L·kg^−1^) obtained from literature databases (e.g., EPI Suite estimates or measured values from published studies; specific values for each OCP congener are provided in Figure S2), and *f*_oc_ = sediment organic carbon content (%) measured in this study (see Supplementary Figure S3).

*PNEC* values were derived using the species sensitivity distribution (SSD) method, with the HC_5_ (concentration protecting 95% of species) taken as the *PNEC*.

Risk levels: no risk (*RQ* < 0.01), low risk (0.01 ≤ *RQ* < 0.1), medium risk (0.1 ≤ *RQ* < 1), high risk (*RQ* ≥ 1).

The combined ecological risk quotient (*RQ*_sum_) for OCP mixtures at each site was calculated as:

$${RQ}_{sum}=\sum_{i=1}^{n}{RQ}_{i}$$

where *RQ_i_* is the risk quotient of the *i*-th OCP compound.

### 2.7. Data Analysis

Spatial distribution maps of OCP concentrations and ecological risks were generated using ArcGIS 10.2 with inverse distance weighting (IDW) interpolation. Principal component analysis (PCA) was performed on OCP concentration data using SPSS 22.0 to identify potential pollution sources. Pearson correlation analysis was used to examine relationships between OCPs and organic matter components.

## 3. Results and Discussion

### 3.1. Regional Characteristics and Comparative Analysis of Organochlorine Pesticide Contamination in Sediments

**Figure 2:**
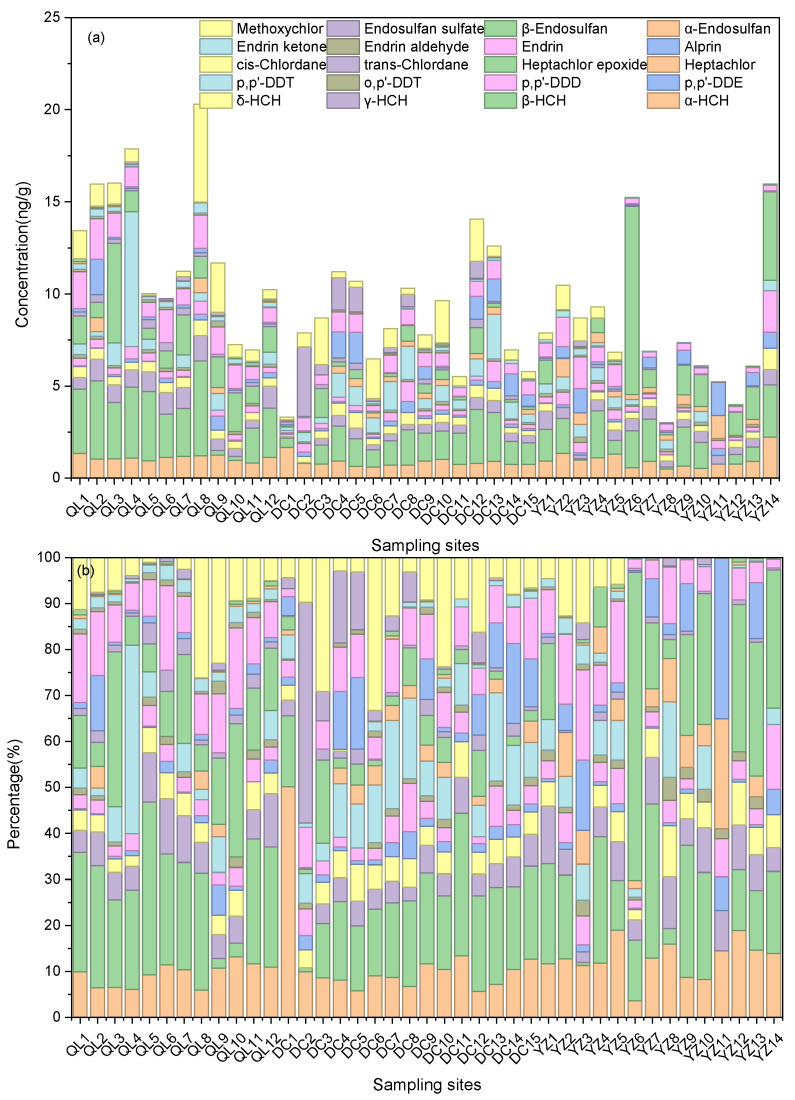
Spatial distribution of concentrations of 20 OCPs in sediments of three plateau lakes: (**a**) absolute concentrations; (**b**) relative concentrations.

Significant differences in the pollution levels of ∑OCPs (20 compounds) in surface sediments were observed among the three plateau lakes (Figure 2a). Qilu Lake exhibited the highest mean ∑OCP concentration at 12.56 ± 4.21 ng/g (range: 6.95–20.30 ng/g), while Dianchi Lake (8.06 ± 2.87 ng/g, range: 3.30–14.06 ng/g) and Yangzonghai Lake (8.07 ± 3.76 ng/g, range: 3.00–15.97 ng/g) showed relatively lower pollution levels, approximately 64% of that in Qilu Lake. This suggests that the Qilu Lake watershed may be subject to stronger historical pesticide input or agricultural non-point source influences, whereas Dianchi Lake and Yangzonghai Lake, despite being located in the same plateau region, exhibit comparatively lower and similar overall OCP contamination levels.

HCHs were once among the most widely used organochlorine pesticides in China; although fully banned since the 1980s, they remain widely distributed due to their environmental persistence [15]. Among the three plateau lakes, Qilu Lake exhibited the highest ∑HCHs contamination level in its sediments (range: 2.06–8.59 ng/g, mean: 5.34 ng/g), while Dianchi Lake (range: 1.16–5.04 ng/g, mean: 3.16 ng/g) and Yangzonghai Lake (range: 1.21–7.04 ng/g, mean: 3.23 ng/g) showed comparable and significantly lower levels (Table 1). Compared with other Chinese lakes, the ∑HCH levels in the three lakes were significantly lower than those in Taihu Lake and Honghu Lake, but higher than those in Chaohu Lake [5,7,15]. When compared with other water bodies, the ∑HCH concentrations in the three lakes were higher than those in inland freshwater aquaculture ponds and the Yangtze River Estuary and adjacent East China Sea, but significantly lower than those in the Yellow River, and were comparable to levels in the mainstream of the Haihe River and the upper and middle reaches of the Huaihe River [21,26,37,38,39,40,41]. In an international context, the ∑HCH levels in the three lakes were close to those measured in Lake Victoria, but significantly lower than those in Ethiopia’s Lake Tana, South Africa’s Lake St. Lucia, and Lake Sibaya [3,11,42]. The watersheds of plateau lakes such as Qilu Lake have historically been agricultural areas dominated by vegetable and flue-cured tobacco cultivation, with a low degree of industrialization and weak point source inputs, resulting in relatively low total residues. HCHs enter the lakes mainly through surface runoff and atmospheric dry/wet deposition [26]. Compared with Taihu Lake, the plateau lakes are surrounded by areas with lower industrialization and weaker point source inputs, leading to lower total residues [7]. Furthermore, the sedimentary environment of the plateau lakes is relatively stable, lacking strong scouring conditions, which allows ∑HCHs to persist at moderate levels [22].

Unlike the contamination pattern of HCHs, the ∑DDT levels in the sediments of the three lakes were generally low (1.09–1.77 ng/g), significantly lower than those in Poyang Lake, Guizhou Caohai Lake, Chaohu Lake, and Honghu Lake, but close to the levels in Baiyangdian Lake [5,6,43,44]. Compared with other water bodies, the ∑DDT concentrations in the three lakes were significantly lower than those in the mainstream of the Haihe River, inland freshwater aquaculture ponds, and the Yellow River, but were close to those in the Yangtze River Estuary and adjacent East China Sea, and slightly higher than those in the middle reaches of the Huaihe River [7,32,38,41]. On an international scale, the ∑DDT levels in the three plateau lakes were far lower than those in all African lakes compared, including Lake Sibaya, Lake St. Lucia, Lake Victoria, and Lake Tana, all of which exhibited contamination levels more than one order of magnitude higher [3,11,41]. This contrasts sharply with the comparison results for HCHs, indicating a substantial difference in historical DDT usage between the southwestern plateau lakes of China and the East African lakes.

The ban on DDTs occurred earlier than that on HCHs, and DDT degradation products (e.g., DDE, DDD) are more readily accumulated under anaerobic conditions [15]. The low DDT levels in plateau lakes such as Qilu Lake can be explained by three main reasons. First, the watersheds are dominated by vegetable cultivation, where the intensity of DDT use is much lower than that in cotton or rice paddies, resulting in limited historical input [17,18,26]. Second, the lake water is slightly alkaline with a high redox potential, which facilitates the degradation of DDTs to DDE and further ring cleavage and mineralization. Third, changes in planting structure in recent years have further reduced new pesticide inputs [21]. Compared with Chaohu Lake and Taihu Lake, the latter were surrounded by extensive rice paddies and industrial areas, leading to greater accumulation of DDT pollution [7,15]. The extremely high DDT residues in Lake Victoria are associated with continued illegal use in some parts of Africa and the long-term input of agricultural runoff into the lake [11].

**Table 1:** Summary of OCP concentrations in surface sediments from three plateau lakes and other regions (ng/g dw).

Location	∑HCHs		∑DDTs		∑Chlors		∑Drins		∑Endosulfans		Reference
	Range	Mean	Range	Mean	Range	Mean	Range	Mean	Range	Mean	
Qilu Lake, China	2.06–8.59	5.34	0.48–8.19	1.77	1.07–5.64	2.08	1.07–4.66	2.00	0.03–0.27	0.15	This study
Dianchi Lake, China	1.16–5.04	3.16	0.36–4.01	1.59	0.10–2.15	0.80	0.19–2.78	1.37	n.d.–3.79	0.68	This study
Yangzonghai Lake, China	1.21–7.04	3.23	0.26–3.69	1.09	0.17–10.55	2.13	0.30–3.50	1.18	n.d.–1.34	0.15	This study
Caohai Lake, China	2.80–37.43	6.53	0.12–47.42	10.86	-	-	-	-	-	-	[6]
Chaohu Lake, China	0.04–7.12	1.12	0.23–85.83	10.53	-	-	-	-	-	-	[44]
Taihu Lake (THB), China	3.3–64.5	13.1	0.2–55.9	8.8	2.0–11.6	3.4	2.6–32.3	6.9	0.7–8.0	1.5	[7]
Taihu Lake (THL), China	3.6–27.8	10.1	1.2–16.5	6.1	2.0–14.4	2.9	1.1–19.5	3.8	0.7–3.2	1.2	[7]
Poyang Lake, China	0.54–6.94	2.95	14.42–82.87	46.69	0–15.34	3.33	n.d.-0.15	0.05	-	-	[16]
Honghu Lake, China	2.54–16.92	7.72	1.84–27.52	9.19	-	-	-	-	-	-	[5]
Baiyangdian Lake, China	1.75–5.70	2.68	0.91–6.48	2.26	-	-	-	-	-	-	[43]
Lake Victoria, Uganda	1.78–4.02	3.30	14.5–385	128	n.d.–2.87	2.30	n.d.–5.56	2.91	1.62–14.6	4.42	[11]
Lake Victoria, Kenya	3.14–3.50	3.17	13.6–138	71.9	n.d.–2.23	2.23	n.d.–0.97	0.91	3.00–3.63	3.19	[11]
Lake Victoria, Tanzania	3.11–3.25	3.19	8.02–239	99.3	n.d.–2.45	2.25	n.d.–2.60	2.39	2.97–275	24.6	[11]
Lake Tana, Ethiopia	10.12–12.66	11.33	33.48–46.37	41.11	-	-	52.11–142.26	85.39	23.09–35.25	30.7	[42]
Lake St Lucia, South Africa	26.3–185.8	82.9	34.5–158.6	87.3	n.d.–70.9	35.1	19.4–171.3	93.6	12.2–126.5	73.2	[3]
Lake Sibaya, South Africa	32.4–176	91.6	61.1–259.9	162.3	5.6–56.3	34.0	48.6–277.5	161.5	16.4–235.1	100.4	[3]
Inland freshwater aquaculture ponds, China	0.22–9.33	0.93	0.13–433	19.7	0.06–17.1	1.02	n.d.–10.3	0.67	1.21–39.1	2.14	[21]
Mainstream of the Haihe River, China	0.71–10.26	2.68	1.54–103.92	28.22	<MDL–0.33	0.05	<MDL–56.84	6.23	<MDL–0.36	0.03	[45]
Upper reach of Huaihe River, China	1.95–11.05	4.53	4.07–23.89	11.07	-	-	-	-	-	-	[37]
Middle reaches of Huaihe River, China	0.88–3.8	1.7	0.017–7.6	0.60	0.019–0.17	0.066	-	-	n.d.–0.67	0.24	[38]
Yangtze River Estuary and the adjacent East China Sea	0.10–0.90	0.34	0.07–4.54	1.75	n.d.–0.23	0.12	-	-	-	-	[40]
Yellow River, China	14.72–29.19	-	6.93–41.87	-	0.94–11.71	-	-	-	5.62–36.79	-	[39]
The coastal East China Sea	0.1–1.6	0.58	1.2–5.6	2.9	-	-	-	-	-	-	[41]
East China Sea	0–0.91	0.20	0–3.61	0.89	-	-	-	-	-	-	[46]
Intertidal zone of eastern China	0.005–5.064	0.378	0.033–4140	19.633	<MDL–0.947	0.085	<MDL–12.884	0.167	<MDL–3.212	0.116	[32]

Notes: Data are presented as range and mean (where available). n.d. = not detected; - = not reported or not analyzed. THB and THL indicate the northern and eastern zones of Taihu Lake, respectively [7]. MDL = method detection limit. Analytical and extraction differences across studies may cause order-of-magnitude variations; results should be interpreted with caution.

It is noteworthy that the three plateau lakes investigated in this study are all closed or semi-closed basins, characterized by water residence times ranging from several years to decades, which substantially prolongs the retention of contaminants within the lacustrine systems [22,25,47]. Concurrently, the relatively low annual mean water temperature (approximately 12–18 °C) in the study region not only suppresses the volatilization and biodegradation of OCPs, but also enhances the sorption of hydrophobic OCPs to sediment organic matter [48,49]. By contrast, eastern plain lakes typically exhibit faster water exchange and higher temperatures, which promote more efficient OCP degradation and result in lower residual concentrations under similar input conditions [7,15]. In addition, the relatively stable hydrodynamic conditions of plateau lakes, marked by weak current scouring, favor the continuous deposition and burial of particle-bound OCPs in sediments [22]. Collectively, the synergistic effects of these three mechanisms account for the observation that, despite lower historical pesticide inputs in the plateau lake watersheds relative to their eastern plain counterparts, moderate OCP residues persist in the sediments of these lakes.

The concentrations of ∑Chlordane in the surface sediments of Yangzonghai Lake (2.13 ng/g) and Qilu Lake (2.08 ng/g) were approximately 2.7 times higher than those in Dianchi Lake. Compared with other Chinese lakes, the ∑Chlordane levels in Yangzonghai Lake and Qilu Lake were close to but slightly lower than those in Taihu Lake and Poyang Lake [7,16], while the levels in Dianchi Lake were comparable to those in Honghu Lake, placing it at a relatively low level among Chinese lakes [5]. In contrast, the ∑Chlordane concentrations in the three lakes were significantly higher than those measured in the mainstream of the Haihe River, the middle reaches of the Huaihe River, the Yangtze River Estuary and its adjacent East China Sea, and the intertidal zone of eastern China [32,37,38,40,45]. On an international scale, the ∑Chlordane levels in Yangzonghai Lake and Qilu Lake were comparable to those in Lake Victoria [11]. In contrast, South Africa’s Lake St. Lucia and Lake Sibaya exhibited extremely high contamination levels [3], which are likely attributable to historical pesticide use or highly concentrated agricultural runoff.

The ∑Drin pollution levels in the three plateau lakes are generally at a relatively low level. Domestically, their concentrations are lower than those in Taihu Lake and the mainstream of the Haihe River [7,45], but higher than those in Poyang Lake, freshwater aquaculture ponds, and the intertidal zone [16,32]. From an international perspective, the ∑Drin concentrations in the three plateau lakes are significantly lower than those in Africa’s Lake Tana, Lake St. Lucia, and Lake Sibaya [3,41], while being relatively close to or slightly lower than the levels in Lake Victoria [11]. The ∑Drin concentrations in the plateau lakes are lower than those in Taihu Lake but higher than those in Poyang Lake [7,16], reflecting regional differences in pesticide usage history among lakes, with the Taihu Lake basin having a relatively higher intensity of aldrin use. In contrast, the extremely high ∑Drin residues in African lakes are primarily associated with intensive agricultural activities in their watersheds [3,41].

This study found that the ∑endosulfan concentration in the surface sediments of Dianchi Lake (0.68 ng/g dw) was significantly higher than that in Qilu Lake and Yangzonghai Lake (both 0.15 ng/g dw). This spatial difference suggests the possible presence of localized point source inputs in Dianchi Lake. Overall, the ∑endosulfan pollution levels in the three plateau lakes are relatively low both domestically and internationally. In comparison, the residues in Taihu Lake and inland freshwater aquaculture ponds are higher, which is closely related to the historically higher intensity of endosulfan use in these regions [7,21]. Pollution in African lakes is particularly severe; for example, the mean concentration in Tanzania’s Lake Victoria is as high as 24.6 ng/g, with a maximum value of 275 ng/g, reflecting substantial differences in pesticide use history and management practices across different regions [11]. Endosulfan residues in the three lakes primarily originate from historical agricultural non-point source inputs, although the local hotspots in Dianchi Lake may also be influenced by additional inputs from industrial or urban drainage. Although China completely banned the use of endosulfan in 2019 [21], due to its persistence, the decline of residues in sediments will be a slow process.

### 3.2. Potential Source Analysis of Organochlorine Pesticides in Sediments

**Figure 3:**
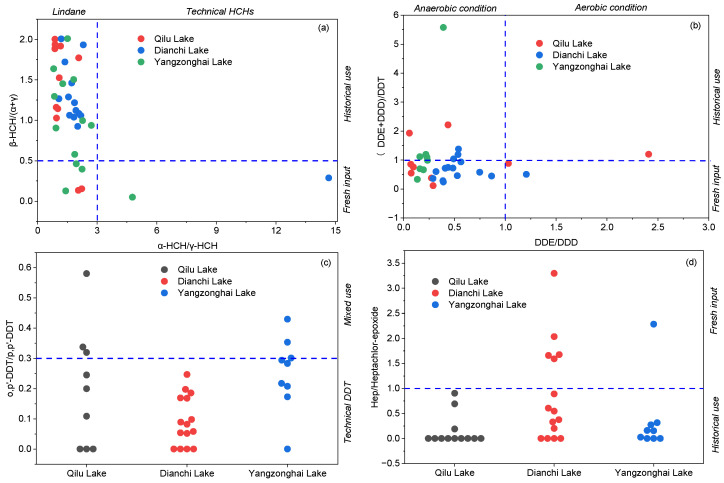
Scatter diagrams of diagnostic ratios of selected OCPs in sediments of three plateau lakes: (**a**) HCHs; (**b**,**c**) DDTs; (**d**) heptachlor and chlordanes.

In this study, the concentrations of β-HCH (0.21–5.16 ng/g) in the sediments of the three lakes were significantly higher than those of α-HCH, γ-HCH, and δ-HCH, accounting for an average of 41.94% of ∑HCHs (Figure 2a,b). Due to its strongest chemical stability and greatest resistance to microbial degradation, β-HCH tends to become relatively enriched during long-term aging, while α-HCH and γ-HCH are more susceptible to degradation or volatilization [7,45,50]. The predominance of β-HCH indicates that HCHs in the three lakes mainly originate from the historical use of technical HCH and have undergone varying degrees of aging [21]. The α-HCH/γ-HCH ratio is a key indicator for distinguishing between technical HCH and lindane sources: a ratio of 3–7 typically indicates continuous technical HCH input, while a ratio below 3 suggests lindane input [6,51]. Except for individual sites in Dianchi Lake (14.67) and Yangzonghai Lake (4.77) where the α-HCH/γ-HCH ratios were anomalously high, indicating technical HCH input, the ratios at the vast majority of sites in the three lakes were below 3 (Figure 3a), suggesting that the lakes are overall influenced by a combination of historical aged residues and lindane input. Furthermore, the β-HCH/(α+γ) ratio can be used to assess the degree of HCH aging, with a ratio greater than 1 generally indicating a higher degree of aging [21]. Except for a few sites near the inlets of Dianchi Lake and Qilu Lake where the β-HCH/(α+γ) ratios were relatively low, the vast majority of sites in these two lakes exhibited ratios greater than 1; in contrast, multiple sites in Yangzonghai Lake showed ratios below 1 (Figure 3a). These findings indicate that although the three lakes are generally dominated by historically aged residues, several localized sites are characterized by relatively fresh HCH inputs or environmental conditions unfavorable for the enrichment of β-HCH.

The spatial heterogeneity of DDTs reflects differences in pollution history, input pathways, and sedimentary environmental conditions among different regions of the three lakes. p,p’-DDT and p,p’-DDD were the dominant components of DDTs in the sediments, accounting for an average of 42.96% and 33.18% of ∑DDTs, respectively (Figure 2a,b). The DDD/DDE ratio is often used to indicate the degradation pathway: a DDD/DDE ratio greater than 1 suggests predominant anaerobic degradation, while a ratio less than 1 suggests predominant aerobic degradation [16,32]. Except for a few individual sites in Qilu Lake and Dianchi Lake where the DDD/DDE ratio exceeded 1, the vast majority of sites across the three lakes exhibited ratios below 1, indicating that the lakes are overall dominated by aerobic degradation, with only localized areas showing anaerobic degradation conditions. Further analysis of the (DDE+DDD)/p,p’-DDT ratio revealed that some sites in Qilu Lake (QL-2, QL-8, QL-9), Dianchi Lake (DC1, DC2, DC3), and Yangzonghai Lake (YZH14) had ratios greater than 1, indicating historically weathered residues. In contrast, the vast majority of sites across the three lakes had ratios below 1, suggesting that the lakes are overall influenced by relatively fresh DDT inputs [16,21,32,38]. Moreover, at multiple sites where p,p’-DDT was not detected, local complete degradation was indicated. Although the production and use of DDT have been banned in China since 1983, the presence of fresh input characteristics suggests the possibility of ongoing or recent sources, such as impurities in dicofol or long-range atmospheric transport [15]. The o,p’-DDT/p,p’-DDT ratios at the vast majority of sites were below 0.3, indicating that the pollution source was historically the use of technical DDT, while ratios above 0.3 at some sites suggest mixed pollution sources [32].

Significant differences were observed in the distribution of heptachlor and its metabolite heptachlor epoxide in the surface sediments of the three lakes. In Qilu Lake, heptachlor was detected only at a few sites, with heptachlor/heptachlor epoxide ratios all below 1, whereas heptachlor epoxide was detected at all sites. This indicates that heptachlor is rapidly transformed into the more stable heptachlor epoxide in the sedimentary environment of Qilu Lake, with historical residues being the primary source [21,38,52]. In Dianchi Lake, heptachlor was widely detected, and heptachlor epoxide was also extensively distributed. At some sites, the hep/heptachlor-epoxide ratio exceeded 1, indicating relatively fresh heptachlor inputs in localized areas [21]. Yangzonghai Lake exhibited a unique distribution pattern: heptachlor was detected alone at some sites without heptachlor epoxide, heptachlor epoxide was detected alone at other sites (up to 10.23 ng/g) without heptachlor, and both compounds were detected at additional sites (hep/heptachlor-epoxide ratios ranging from 0.03 to 2.28). This reflects a high degree of spatial heterogeneity in heptachlor inputs and degradation/transformation in this lake.

Regarding chlordane compounds, trans-chlordane was widely detected in all three lakes (n.d.–0.46 ng/g). Notably, cis-chlordane was detected only at one site in Dianchi Lake and was absent from all other sites. Although technical chlordane typically contains approximately equal proportions of cis-chlordane (13%) and trans-chlordane (11%), and cis-chlordane degrades slightly faster than trans-chlordane in the environment [4], the nearly complete absence of cis-chlordane alongside the persistent and widespread presence of trans-chlordane observed in this study is more likely related to the specific isomer composition of the chlordane products historically used in this region.

In the surface sediments of the three lakes, endrin was the absolute dominant component among aldrin-related compounds. Endrin was widely detected with relatively high concentrations in Qilu Lake, while localized sites in Yangzonghai Lake exhibited characteristics of fresh aldrin input [18,21]. The concentrations of endosulfan compounds were generally low, although relatively higher levels of endosulfan sulfate were observed at some sites in Dianchi Lake, indicating localized input or accumulation of degradation products [7,21]. Methoxychlor showed relatively high detection rates and significant spatial variability across all three lakes, suggesting new environmental inputs of methoxychlor in localized areas.

**Figure 4:**
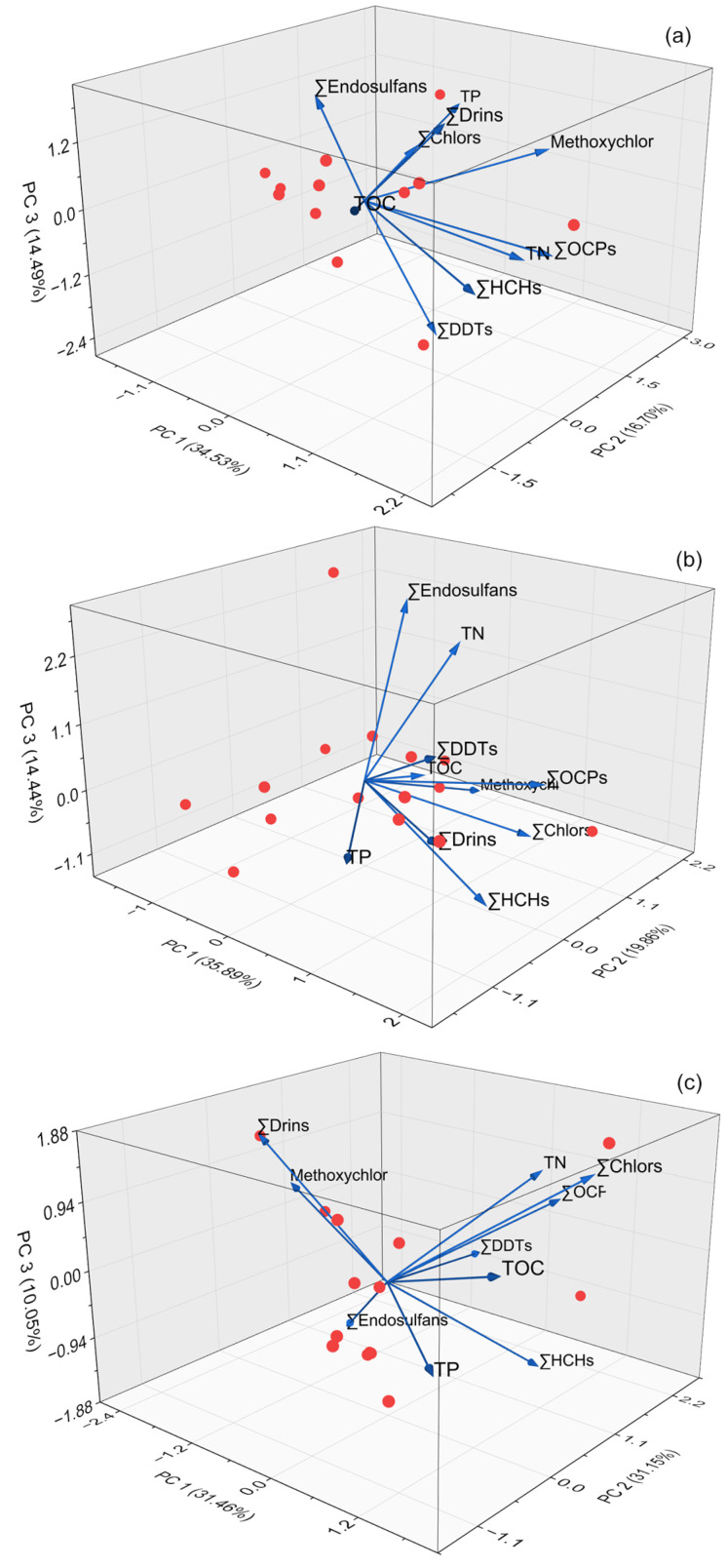
Principal component analysis (PCA) of OCP concentrations and sediment properties in three plateau lakes: (**a**) Qilu Lake, (**b**) Dianchi Lake, (**c**) Yangzonghai Lake. The red dots represent sampling sites.

In this study, principal component analysis (PCA) was used to identify the sources of OCPs. The PCA procedure comprised data standardization, the use of a correlation matrix, and the retention of principal components determined by the eigenvalue > 1 criterion, in conjunction with the inflection point of the scree plot. Total nitrogen (TN) and total phosphorus (TP) were incorporated as supplementary variables, serving as proxies for agricultural runoff intensity, given that fertilizer application in the surrounding agricultural areas constitutes a common source of both nutrients and OCPs via farmland surface runoff [6,32]. Three principal components were extracted for Qilu Lake, Dianchi Lake, and Yangzonghai Lake, cumulatively explaining 65.72%, 70.19%, and 72.66% of the total variance, respectively (Figure 4). Although the value does not reach 80%, this level of explanation is commonly reported in environmental source apportionment studies that involve multiple variables and confounding factors [11,32]. For Qilu Lake, PC1 (34.53%) showed high positive loadings of ∑HCHs, ∑OCPs, TN, and ∑Drins, indicating that a mixed source dominated by HCHs and Drins is associated with total nitrogen, reflecting the synergistic impact of agricultural non-point source inputs on nitrogen and pesticide residues [11,32]; PC2 (16.70%) exhibited high positive loadings of methoxychlor and TP, while TOC showed a negative loading, indicating that the association of methoxychlor with phosphorus is influenced by organic matter distribution; PC3 (14.49%) displayed positive loadings of ∑Endosulfans, ∑Drins, and ∑Chlors along with TOC, reflecting the co-accumulation of historical residues or degradation products [45].

For Dianchi Lake, PC1 (35.89%) showed high positive loadings of ∑HCHs, ∑Drins, ∑Chlors, ∑DDTs, ∑OCPs, and TP, representing a mixed source of multiple OCPs closely associated with phosphorus, indicating agricultural or urban non-point source inputs [6,32]; PC2 (19.86%) exhibited positive loadings of methoxychlor and ∑Chlors along with TN, while TP showed a negative loading, indicating their association with nitrogen and a source different from that of phosphorus; PC3 (14.44%) exhibited extremely high positive loadings of ∑Endosulfans and TN, along with a positive correlation of ∑DDTs, reflecting the association of endosulfans and DDTs with nitrogen, which may be related to specific pesticide use histories. For Yangzonghai Lake, PC1 (31.46%) showed positive loadings of ∑Chlors, TOC, and TP, while negative loadings of ∑Drins, methoxychlor, and ∑Endosulfans were observed, indicating that chlordanes are positively associated with organic matter and phosphorus and are distinguished from other OCPs, reflecting their unique input history [15,39,45]; PC2 (31.15%) exhibited very high positive loadings of ∑OCPs, ∑DDTs, ∑HCHs, and TN, representing a mixed source dominated by HCHs and DDTs closely associated with nitrogen, indicating the dominant role of agricultural non-point source inputs; PC3 (10.05%) exhibited positive loadings of ∑Drins, ∑Chlors, and TN, while negative loadings of ∑HCHs and ∑Endosulfans were observed, reflecting the association of Drins and chlordanes with nitrogen and indicating differences in sources or degradation behaviors from HCHs and endosulfans.

### 3.3. Potential Ecological Risk Assessment of Organochlorine Pesticides in Sediments

**Figure 5:**
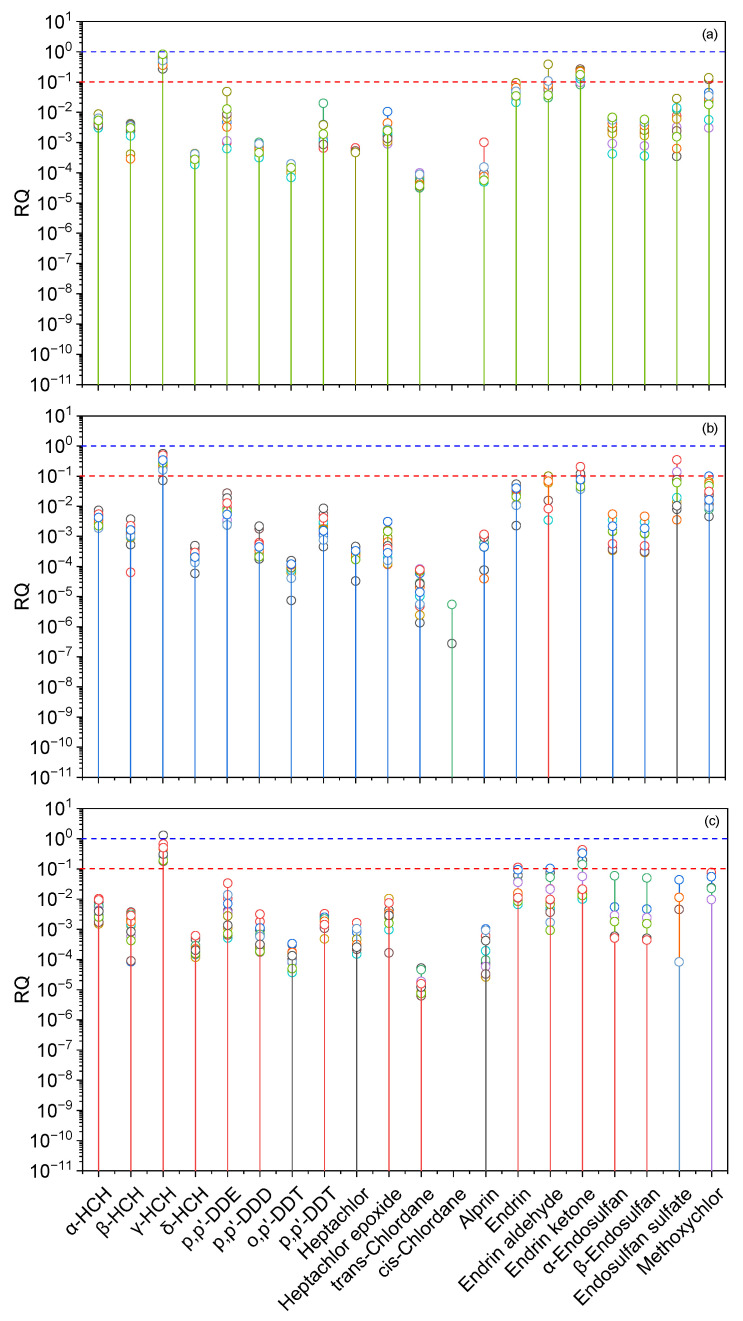
Risk quotients (RQs) of OCPs in sediments of three plateau lakes: (**a**) Qilu Lake, (**b**) Dianchi Lake, (**c**) Yangzonghai Lake. Circle colors represent sampling sites; line colors represent lakes.

**Figure 6:**
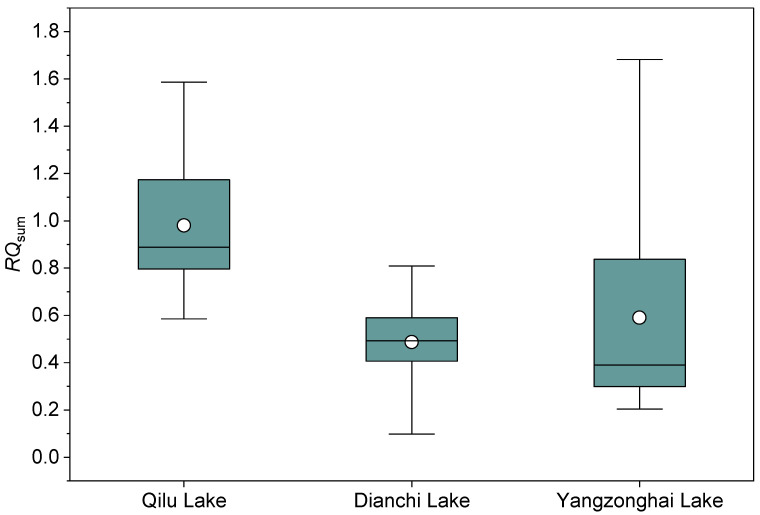
Total risk quotients (*RQ*_s_) for OCPs in sediments of three plateau lakes (combined *RQ*_s_ for all OCP congeners). The white circles represent the mean values.

Based on the risk quotient (*RQ*) classification criteria from the European Union Technical Guidance Document, this study conducted a systematic assessment of the individual and combined ecological risks of organochlorine pesticides (OCPs) in the surface sediments of three lakes (Figure 5a–c and Figure 6). This method is a common approach for assessing the ecological risk of pesticides in aquatic sediments, where the *RQ* value is calculated by dividing the measured concentration (MC) in sediment samples by the predicted no-effect concentration (*PNEC*) [41,53]. In this study, the RQ was further refined using porewater concentrations derived from the equilibrium partitioning method. This approach accounts for the bioavailability of OCPs in sediments, as only the dissolved fraction in porewater is considered to pose a potential ecological risk to benthic organisms. The measured foc values for each sampling site (Supplementary Figure S3; Section 2.6) were incorporated into this calculation. According to existing studies, the ecological risk levels are classified as follows: *RQ* > 1 indicates high risk, 0.1 < *RQ* < 1 indicates medium risk, and *RQ* < 0.1 indicates low risk [2]. Fish, daphnia, and algae are commonly used as assessment receptors to accurately reflect the potential impact of pesticide residues on aquatic ecosystems [41]. The assessment results showed that for most OCP compounds (e.g., α-HCH, β-HCH, δ-HCH, DDTs and their degradation products, heptachlor, heptachlor epoxide, chlordanes, aldrin, endosulfans, etc.) in the three lakes, the *RQ* values were below 0.01, indicating no risk or low-risk levels. The residual concentrations of these compounds are extremely low and do not yet pose a significant ecological threat to benthic organisms [39,45]. Pesticides are mostly hydrophobic compounds that tend to stabilize in sediments after undergoing a series of environmental chemical behaviors in water; therefore, the ecological risks of pesticide residues in sediments warrant attention [7,32]. The low-risk levels of most OCPs in this study indicate that the accumulation of these compounds in sediments has not yet reached a level that would cause obvious adverse effects on aquatic organisms.

However, γ-HCH (lindane) was a ubiquitous major risk contributor across the three lakes. In Qilu Lake, γ-HCH *RQ* values at all sites ranged from 0.27 to 0.84, all reaching medium-risk levels, with site QL-2 (0.84) approaching the high-risk threshold, which is consistent with the findings from water body studies of this lake [21]. In Dianchi Lake, γ-HCH *RQ* values at some sites ranged from 0.17 to 0.38, indicating medium risk. In Yangzonghai Lake, γ-HCH *RQ* values at most sites ranged from 0.18 to 0.51, with site YZ1 (1.30) exceeding the high-risk threshold, indicating a significant ecological risk at this site. The relatively low *PNEC* value and high toxicity of γ-HCH are likely the main reasons for its elevated *RQ* values [21,54]. It is worth noting that the risk distribution of γ-HCH exhibited clear spatial heterogeneity across the three lakes. All sites in Qilu Lake reached medium-risk levels, a high-risk site appeared in Yangzonghai Lake, while the overall risk in Dianchi Lake was relatively low. This spatial heterogeneity may be partly attributable to the variability in foc across sampling sites, as the equilibrium partitioning calculation explicitly accounts for foc, thereby influencing the derived porewater concentrations and *RQ* values. A significant negative correlation (Figure S4, *p* < 0.01) was observed between foc and *RQ*_sum_ values, indicating that in sediments with higher organic carbon content, hydrophobic OCPs are more strongly sorbed to the solid phase, resulting in lower dissolved concentrations in porewater and thus reduced bioavailability and potential ecological risk. In other words, sediments with high organic matter content serve as a “buffer” against ecological risk by enhancing the solid-phase retention of OCPs. This finding underscores the importance of incorporating foc parameters into ecological risk assessments for plateau lake sediments; otherwise, neglecting the spatial variability of foc may lead to overestimation or underestimation of the actual bioavailability of OCPs. These findings indicate that although the ecological risks of most OCPs in the three lakes are low, the widespread presence of γ-HCH and its distribution characteristics at medium- and high-risk sites warrant significant attention.

Endrin and its derivatives also exhibited non-negligible risk levels in the three lakes. Endrin showed *RQ* values ranging from low risk to the boundary of medium risk in Qilu Lake, and reached medium risk at site YZ2 (0.11) in Yangzonghai Lake. Endrin aldehyde reached medium risk at site QL-9 (0.39) in Qilu Lake and at site YZ3 (0.10) in Yangzonghai Lake. Endrin ketone reached medium risk at all sites in Qilu Lake except QL-4 (0.08), and reached medium risk at sites YZ1 (0.19), YZ2 (0.43), and YZ3 (0.33) in Yangzonghai Lake. In addition, methoxychlor reached medium risk at sites QL-8 (0.12) and QL-9 (0.14) in Qilu Lake. Considering the relatively low recovery of methoxychlor (71%), we performed a sensitivity analysis by applying a recovery correction factor (corrected concentration = measured concentration/recovery). The corrected RQ values at sites QL-8 and QL-9 remained within the medium-risk category. Endosulfan sulfate reached medium risk at sites DC2 (0.35), DC4 (0.13), and DC5 (0.14) in Dianchi Lake, indicating localized input or accumulation of degradation products. It is recommended that further source tracing investigations and continuous monitoring be carried out for the high-risk sites (especially Yangzonghai Lake YZ1 and Qilu Lake QL-9) as well as the sites with elevated endosulfan sulfate levels in Dianchi Lake.

In terms of the combined ecological risk entropy (*RQ*_sum_), Qilu Lake exhibited *RQ*_sum_ values ranging from 0.58 to 1.59, with seven sites at medium-risk levels (0.1 ≤ *RQ*_sum_ < 1) and five sites at high-risk levels (*RQ*_sum_ ≥ 1). Dianchi Lake had *RQ*_sum_ values ranging from 0.10 to 0.81, generally at low to medium risk levels, with no high-risk sites. Yangzonghai Lake exhibited *RQ*_sum_ values ranging from 0.20 to 1.68, with most sites at medium-risk levels, among which YZ1 (1.68) and YZ2 (1.35) reached high-risk levels. The high-risk sites may be related to local historical pesticide use, riverine inputs, or differences in sedimentary dynamic conditions.

In summary, γ-HCH was a ubiquitous risk contributor across the three lakes, particularly prominent in Qilu Lake and Yangzonghai Lake. Endrin and its derivatives (endrin aldehyde, endrin ketone), as well as methoxychlor, further elevated the combined risk levels at localized sites. The overall ecological risk level of Dianchi Lake was lower than that of Qilu Lake and Yangzonghai Lake. The incorporation of foc into the equilibrium partitioning-based RQ calculation ensures that the risk assessment accounts for site-specific sediment properties, thereby providing a more environmentally realistic evaluation of OCP bioavailability and ecological risk.

## 4. Conclusions

This study systematically investigated the contamination characteristics, sources, and ecological risks of OCPs in surface sediments of three typical plateau lakes in Yunnan Province. HCHs were identified as the dominant contaminants, whereas DDTs exhibited generally low concentrations. Source apportionment revealed that agricultural non-point source pollution was the primary source of OCP residues, although localized point source inputs were indicated by elevated endosulfan levels in Dianchi Lake and increased chlordane concentrations in Yangzonghai and Qilu lakes. Ecological risk assessment demonstrated that γ-HCH (lindane) ubiquitously presented moderate risk across all lakes, with site YZ1 in Yangzonghai Lake exceeding the high-risk threshold. The comprehensive ecological risk, expressed as *RQ*_sum_, was highest in Qilu Lake (0.58–1.59), followed by Yangzonghai Lake (0.20–1.68), and lowest in Dianchi Lake (0.10–0.81). Accordingly, enhanced source tracing and risk control measures should be prioritized in areas with elevated risk levels.

## Data Availability

The raw data supporting the conclusions of this article will be made available by the authors upon request.

## Supplementary Materials

The following supporting information can be downloaded at: https://www.mdpi.com/article/10.3390/toxics14070556/s1, Figure S1: Distribution of (a) total organic carbon (TOC), (b) total nitrogen (TN), and (c) total phosphorus (TP) contents in surface sediments. Figure S2: Organic carbon-normalized partition coefficients (*K*_oc_) of the 20 target OCP congeners used in the equilibrium partitioning method (values obtained from literature databases, including EPI Suite estimates and measured values from published studies); Figure S3: Distribution of sediment organic carbon content (foc, %) at each sampling site in Qilu Lake, Dianchi Lake, and Yangzonghai Lake. Figure S4: Correlation analysis between sediment organic carbon content (foc, %) and the combined ecological risk quotient (*RQ*_sum_) of OCPs at each sampling site in Qilu Lake, Dianchi Lake, and Yangzonghai Lake.
