# Supplementary material for: Occurrence, Sources, and Ecological Risks of Organochlorine Pesticides in Sediments of Typical Plateau Lakes, Southwest China
Source: Toxics. 2026 Jun 25;14(7):556. doi: 10.3390/toxics14070556 (PMC13416866; doi:10.3390/toxics14070556)
Supplement: Supplementary file 1 [file toxics-14-00556-s001.zip › toxics-4346398-supplementary.pdf]

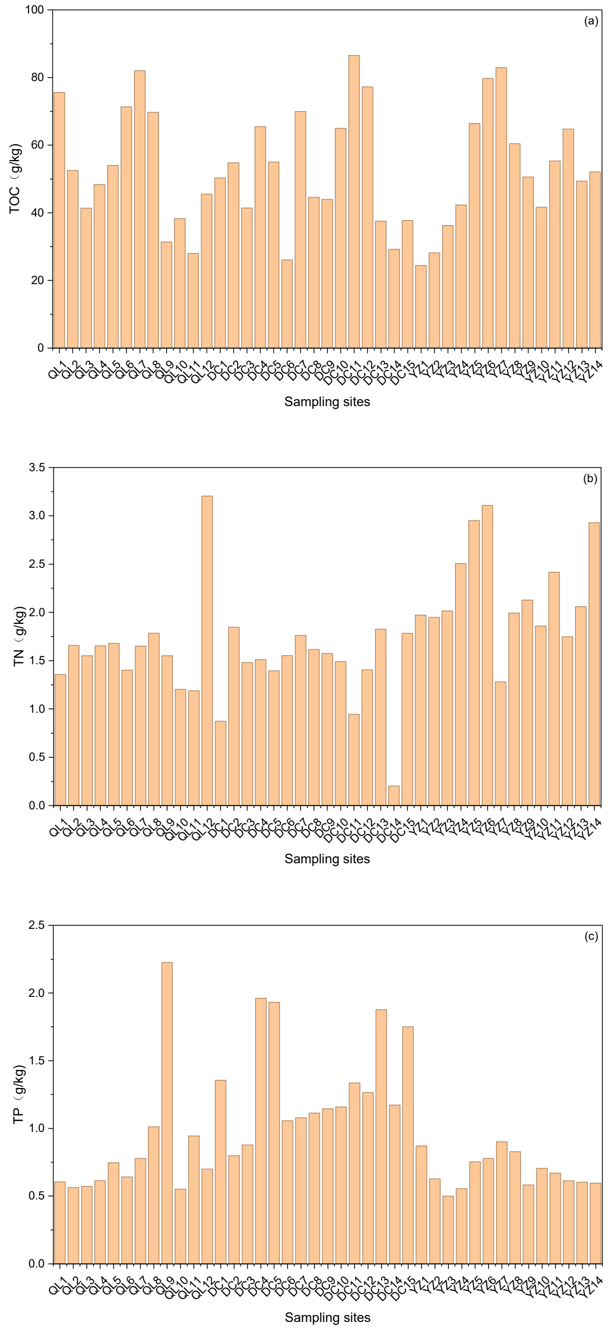

Fig. S1 Distribution of (a) total organic carbon (TOC), (b) total nitrogen (TN), and (c) total phosphorus (TP) contents in surface sediments.

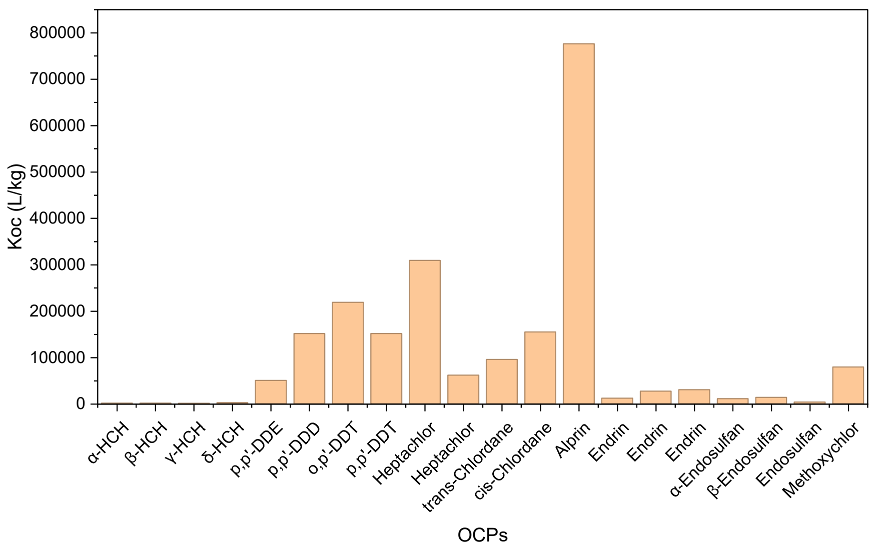

Fig. S2 Organic carbon-normalized partition coefficients (Koc) of the 20 target OCP congeners used in the equilibrium partitioning method (values obtained from literature databases, including EPI Suite estimates and measured values from published studies).

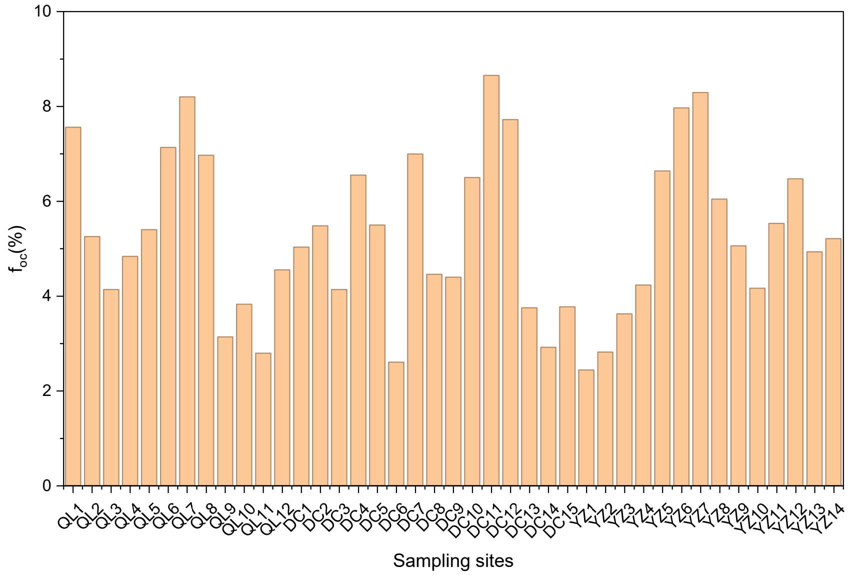

Fig. S3 Distribution of sediment organic carbon content (foc, %) at each sampling site in Qilu Lake, Dianchi Lake, and Yangzonghai Lake

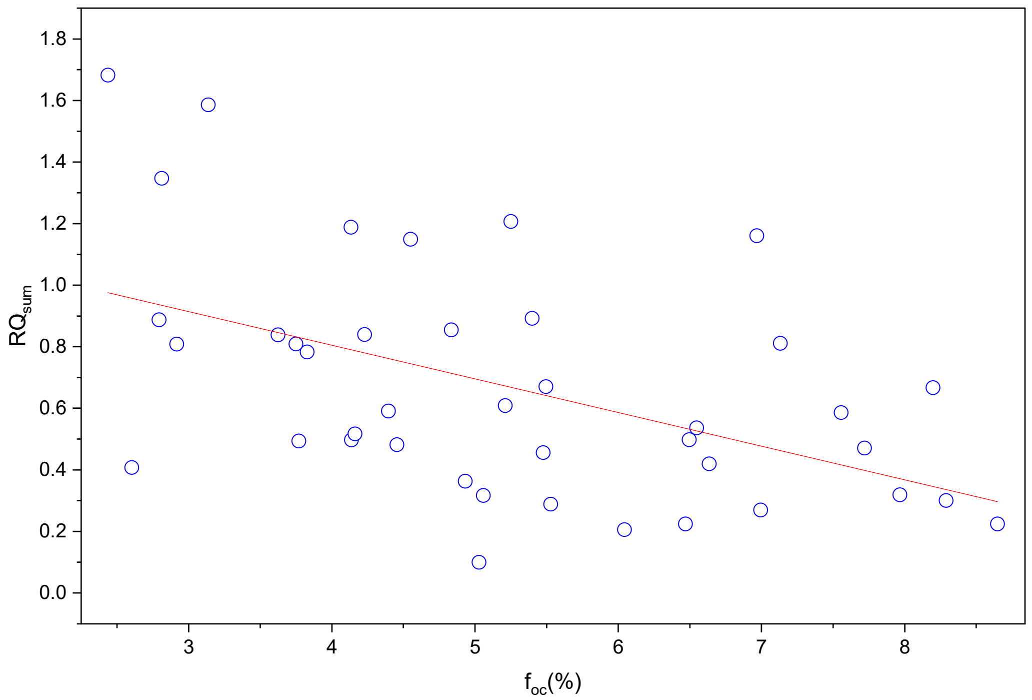

Fig. S4 Correlation analysis between sediment organic carbon content (foc, %) and the combined ecological risk quotient (RQ_{sum}) of OCPs at each sampling site in Qilu Lake, Dianchi Lake, and Yangzonghai Lake
